# Epidemiological and clinical characteristics of lung cancer in Saudi Arabia: a retrospective study in single oncology center

**DOI:** 10.32604/or.2024.052358

**Published:** 2024-10-16

**Authors:** YOUSEF KATIB, NASSER MULLA

**Affiliations:** 1 Department of Radiology, College of Medicine, Taibah University, Madinah, 42353, Saudi Arabia; 2 Department of Internal Medicine, College of Medicine, Taibah University, Madinah, 42353, Saudi Arabia

**Keywords:** Lung cancer (LC), Epidemiology, Smoking, Genetic mutations, Saudi Arabia

## Abstract

**Background:**

Lung cancer (LC) is one of the most common neoplastic diseases and a leading cause of death in Saudi Arabia. Its incidence in Saudi Arabia has increased by more than 3% within two decades. Our study aimed to describe the epidemiological and genetic landscapes of LC in Al-Madinah city in Saudi Arabia.

**Methods:**

A retrospective analysis was conducted on the medical records of 65 patients diagnosed with lung cancer between 2015 and 2021 at a single medical oncology center in Al-Madinah city of Saudi Arabia.

**Results:**

The mean patients’ age was 59.2 years, with 50 (76.9%) males and 15 (23.1%) females; 37 (57%) smokers, and 28 (43%) non-smokers. The number of cases per year has increased gradually over six years from 2015 (n = 3) to 2020 (n = 13). The most prevalent histopathological diagnosis was non-small cell lung cancer (NSCLC) (n = 58, 89%) followed by small cell lung cancer (SCLC) (n = 5, 7.8%). NSCLC was frequently more common in smokers while squamous cell carcinoma was more frequent in non-smokers. Around 89% (n = 58) of the cases were diagnosed in late stage IV and the most common metastatic sites were to pleura and lymph nodes (n = 32, 49.2%). Program Death Legend-1 (*PDL-1*) was fairly expressed in 7/10 (70%) patients. Epidermal Growth Factor Receptor (*EGFR*) was mutated in 5/17 (29%) patients. Other mutations detected include Anaplastic Lymphoma Kinase (*ALK*) and phosphatidylinositol 3-kinase (*PIK3C*) mutations in two patients.

**Conclusions:**

Our study revealed that lung cancer is a significant burden in Al-Madinah city of Saudi Arabia. If the risk factors are not controlled, the number of cases may increase considerably. Health education about the risk factors and cancer prevention helps in early lung cancer detection.

## Introduction

Lung cancer (LC) is a leading cause of cancer-related mortality worldwide [1]. It ranks as the second most commonly diagnosed cancer type, following breast cancer, and its incidence has been on the rise over the past decade [2]. In the United States, approximately 230,000 individuals are diagnosed with lung cancer each year, with an estimated annual mortality rate of around 135,000 patients [3,4]. In 2024, 234,580 new lung cancer cases and 125,070 cancer deaths are projected to occur in the United States. The decline in cancer mortality has been ongoing, with over a hundred thousand deaths averted since 1991 due to factors such as reduced smoking rates, improved early detection methods, and advancements in treatment options for both early and advanced stages of cancer. Among all cancer types in the United States, the highest number of deaths are attributed to lung, colorectal, and pancreatic cancers. Approximately 20,300 lung cancer deaths not related to smoking would rank as the eighth leading cause of cancer death if classified separately. The trends in cancer incidence and mortality have been extensively documented by the American Cancer Registry for 2024. Therefore, it is crucial for healthcare systems to prioritize global efforts aimed at preventing lung cancer and improving the prognosis for diagnosed patients. One crucial aspect of lung cancer prevention is addressing the risk factors associated with the disease. Lung cancer is considered multifactorial, with tobacco smoking being the most significant risk factor [3]. The exposure to second-hand smoke is also recognized as a contributing factor, with approximately 23% of non-smokers reporting exposure to second-hand smoke within the past seven days [3]. Occupational exposure to carcinogens like radon and asbestos is another identified risk factor for lung cancer. Exposure to silica, nickel, arsenic, and beryllium has also been linked to an increased risk of developing lung cancer [3].

Smoking was prohibited in Saudi Arabia under religious law until the 1960s. Although it has since become legal, smoking remains culturally unacceptable. However, the incidence of lung cancer has posed a significant economic burden in Saudi Arabia since its legalization. It has been estimated that the high prevalence of lung cancer has had a negative impact on quality of life and productivity. Out of the 166,497 new cancer cases reported between 1999 and 2013, 3.8% were lung cancer cases [5]. Males and Saudi nationals had more than three times the number of cases compared to females and non-Saudis, respectively. The Saudi Cancer Registry (SCR) has observed a continuous increase in lung cancer cases in Saudi Arabia since the 20th century [1]. In 2014, the SCR recorded 452 cases of lung cancer, which is considered a low incidence rate compared to international standards. These cases accounted for 3.9% of all reported cancer cases that year. In a survey conducted in 2016, the prevalence of smoking in males was reported to be 29%, while in females it was only 2% [3]. As a result, Saudi Arabia adopted the WHO Framework Convention on tobacco control in 2015, leading to the implementation of a smoking ban in public places. In addition to that, cigarette taxation was introduced in 2017. Despite the local rates remaining relatively stable, the global incidence of lung cancer has significantly increased, especially with the projected growth of the elderly population. In 2018, the incidence rate of lung cancer in Saudi Arabia was approximately 3.8%, with a mortality rate of 7.4% according to SCR [6]. Fortunately, in 2019–2020, the number of diagnosed cases remained consistent at 458, maintaining the same incidence rate compared to international rates [6]. These cases accounted for 3.4% of all reported cancer cases that year. A retrospective cohort analysis conducted by Almatroudi et al. examined 4530 lung cancer patients in Saudi Arabia between 2006 and 2016. The study revealed an overall age-standardized incidence-sex ratio of 3.2 per 100,000 cases. The incidence of lung cancer increased with age, with a reported rate of 23% in the age group of individuals over 75 years old [7].

Research studies examining the epidemiological, genetic, and histological aspects of lung cancer in the Al-Madinah region of Saudi Arabia have been underestimated. In a study conducted by Albasri in 2019, an 11-year retrospective analysis was performed to identify the most prevalent subtypes of lung cancer in Al-Madinah city [8]. The findings revealed that lung adenocarcinoma was the most common subtype, accounting for 47% of cases, followed by squamous cell carcinoma at 25%, along with other types of cancers [1].

The National Lung Cancer Screening Trial demonstrated that screening for lung cancer among high-risk individuals can reduce mortality by 20% [6]. However, the guidelines issued by the Saudi Lung Cancer Association (SLCA) and Saudi National Cancer Center did not recommend mass screening for lung cancer as a national program. Instead, they provided guidance to healthcare professionals considering lung cancer screening for very high-risk patients [4]. This decision was influenced by the limited number of cases in Saudi Arabia and the absence of a cost-effectiveness analysis regarding the feasibility of conducting computed tomography (CT) scans in the country.

Surgery remains the primary treatment option for early-stage NSCLC, with the goal of curative intent even after intraoperative confirmation of the disease stage. The choice of management approach is determined by the stage of lung cancer at the time of diagnosis. Following surgery, the patient’s best treatment approach is determined based on tumor staging, grading, and genetic findings, which may involve radiotherapy, chemotherapy, targeted therapy, or a combination of these modalities. In Saudi Arabia, there are more than 30 thoracic surgeons and 13 radiotherapy centers located throughout the Kingdom to provide post-surgical treatment for lung cancer patients [6]. The SLCA, in collaboration with the Saudi National Cancer Center, has developed guidelines specifically tailored to our patient population, taking into account patients’ characteristics, disease biology, and practice settings. Genetic testing for lung cancer is considered an important targeted approach, both before and after surgery and radiotherapy. Despite the advancements in understanding the genetic landscape of lung cancer, genetic panels are commonly performed in Saudi Arabia but are rarely reported, and no reports have been published specifically for the Al-Madinah region.

Our study aims to explore some epidemiological and genetic landscapes of lung cancer patients in Al-Madinah city in Saudi Arabia, between the years 2015 and 2021, which has not been previously investigated since the 20th century.

## Methods

### Cases stratification

This research study was approved by the Institutional Review Board of the General Directorate of Health Affairs in Al-Madinah city in Saudi Arabia (no. 2019–5161). Patients’ consents were not required for this study. The study included 65 patients diagnosed and treated in the Medical Oncology Center at King Fahad Hospital in Al-Madinah City of Saudi Arabia within the period from 2015 to 2021. All enrolled cases in this research study were above 18 years old, diagnosed clinically and histologically with lung cancer of different types and staging, and included both the Saudi population and Expatriates who were living and working in Saudi Arabia. Clinical information including age, year of the diagnosis, sex, nationality, major clinical presentation, pathology report, and treatment plan were obtained from the hospital records. Patients’ pathology report contained the patient’s biopsy site and specific histological diagnosis including tumor histological subtype (Non-small cell lung cancer (NSCLC), Large cell adenocarcinoma, Mucoepidermoid carcinoma, and Small cell lung cancer (SCLC)), TNM staging, and the site of metastasis. Information regarding genetic mutation was also obtained from the medical record. Next-generation sequencing (NGS) and Immunohistochemistry (IHC) from the paraffin-embedded tumor tissue to identify specific mutations-related treatment in lung cancer were retrospectively performed. All patients underwent standard treatment protocol for lung cancer based on the TNM staging and genetic findings. All information is summarized in Table 1.

**Table 1:** Characteristics of the study population in lung cancer cases between 2015–2021

	N	%
**Age,** mean (SD)	59.2 (12.5)	
**Sex**		
Female	15	23.1
Male	50	76.9
**Smoking**		
Non-smoker	28	43.0
Smoker	37	57.0
**Nationality**		
Saudi	42	64.5
Non-Saudi	23	35.5
**Year diagnosis**		
2015	3	4.6
2016	7	10.8
2017	11	16.9
2018	17	26.2
2019	13	20.0
2020	13	20.0
**Site of the biopsy**		
Lung biopsy	47	72.4
Biopsy from metastatic site	18	27.6
**Major clinical presentation**		
Cough	51	78.5
Shortness of breath (SOB)	44	67.7
Chest pain	23	35.4
Weight loss	7	10.8
Shoulder pain	2	3.1
**Histopathological diagnosis**		
NSCLC	58	89.0
Large cell neuroendocrine carcinoma	1	1.6
Mucoepidermoid carcinoma	1	1.6
SCLC	5	7.8
**TNM staging**		
I, II, III	7	10.8
IV: Metastasis	58	89.2
**Metastasis site**		
Pleura, pleural effusion	19	17.7
Lymph nodes	13	12.1
Liver	17	15.8
Bone	26	24.4
Kidney	2	1.9
Brain	13	12.1
Adrenal gland	11	10.4
Missing	6	5.6
**Non-surgical treatment**		
Chemotherapy	28	43.0
Immunotherapy	4	6.2
Targeted therapy	3	4.6
Palliative	26	40.0
Not applicable	4	6.2
**Number of lines of therapy received**		
1	20	69.0
2	7	24.1
≥3	2	6.9
**EGFR**: *Only 17 patients tested*		
Mutant	4	29.4
with Tumor Protein 53 (TP-53)	1	
Wildtype	12	70.6
**PD-L1**: *Only 10 patients tested*		
High expression	4	40.0
Low expression don	3	30.0
Lack of expression	5	50.0
**Other mutations**		
ALK	1	1.5
TP53	2	3
PIK3CA	1	1.5
Failed	4	6.1
Negative	3	4.6
Not performed	49	75.3

### Statistical analysis

Descriptive data were presented as frequencies and percentages. The Fisher-exact test was used to assess the association between categorical variables. The statistical analyses were performed using IBM SPSS ver. 24 and SigmaPlot, Systat software program version 12 (SPSS Inc., Chicago, IL, USA).

## Results

**Figure 1:**
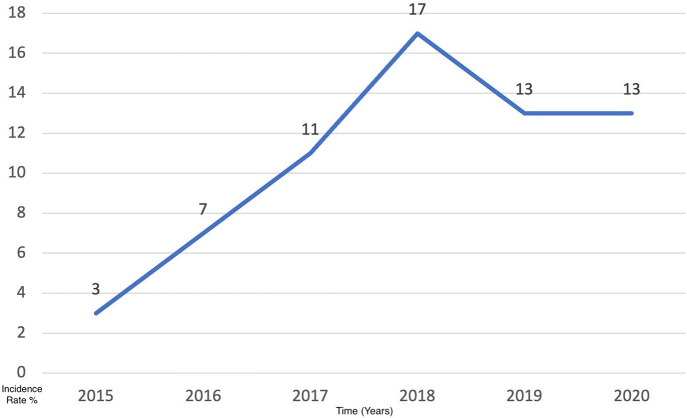
The incidence rate of lung cases per year within 6 years from 2015 to 2020.

The results of this study have been analyzed for the 6-year period [2015–2021]. The mean patients’ age was 59.2 years (SD: 12.5), with 50 (76.9%) males and 15 (23.1%) females. The majority of patients were Saudi nationals (n = 42, 64.4%) whose ages ranged between 47–71 years. Out of those patients, 37 (57%) were smokers and 28 (43%) were non-smokers. The number of cases per year has increased gradually throughout the 6 years from 2015 (n = 3) to 2020 (n = 13) (Table 1, Fig. 1).

**Figure 2:**
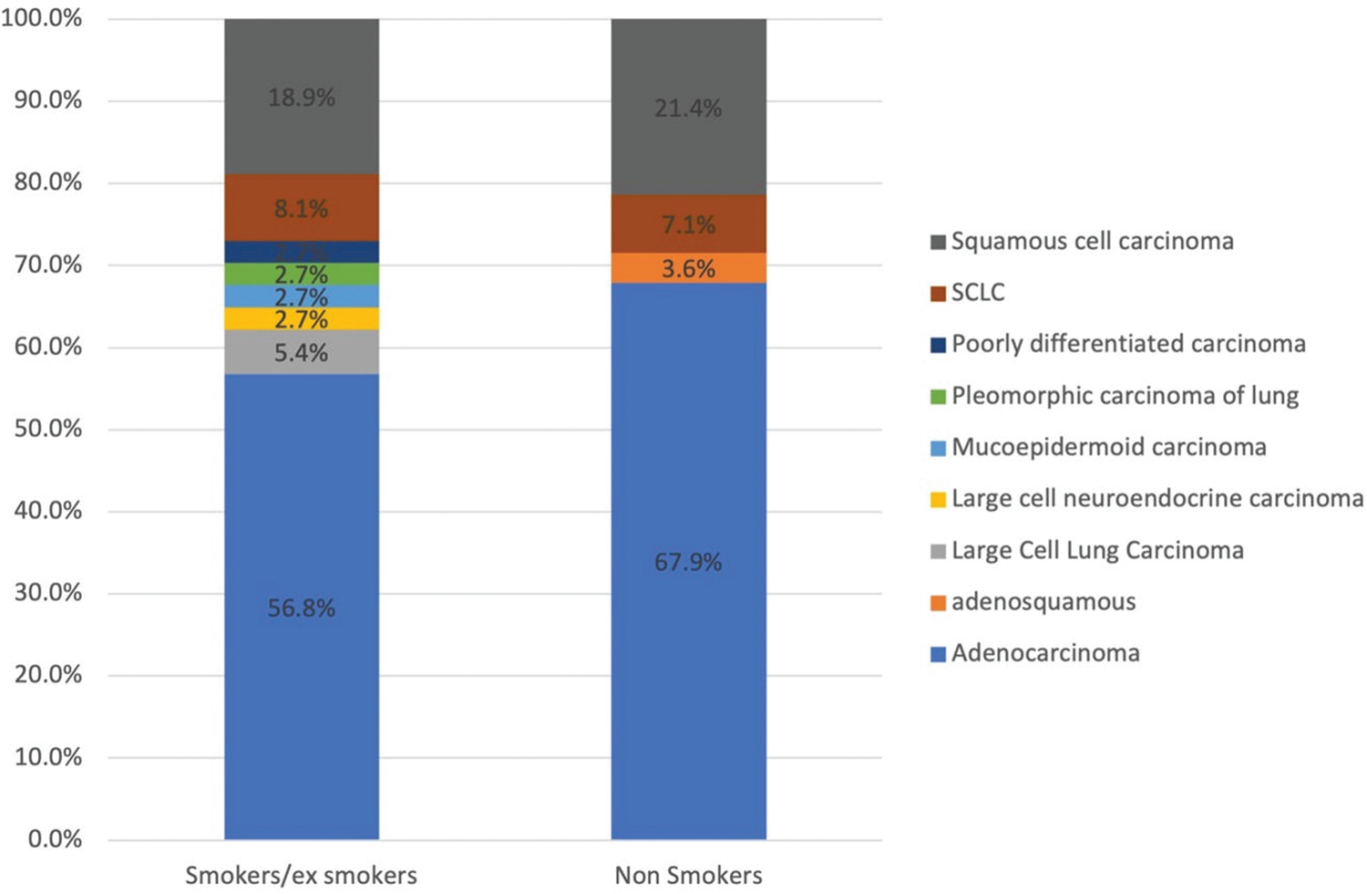
The distribution of lung cancer subtypes among smokers and non-smokers.

The main clinical presentation was repetitive cough (n = 51, 78.5%) followed by shortness of breath (n = 44, 66.7%). Other symptoms included chest pain, weight loss, and shoulder pain. The biopsy diagnosis from the lung was established in 72.4% (n = 47) of the patients compared to a diagnosis established from metastatic deposits in 27.6% (n = 18) of the cases. The most prevalent histopathological diagnosis was NSCLC (n = 58, 89%) followed by SCLC (n = 5, 7.8%), large cell neuroendocrine carcinoma (n = 1, 1.6%), and mucoepidermoid carcinoma (n = 1, 1.6%). There was an insignificant statistical difference in lung cancer subtypes among smokers and non-smokers however, NSCLC was frequent in smokers while squamous cell carcinoma was more common in non-smokers (Fig. 2).

Around 89% (n = 58) of the cases were diagnosed in late-stage IV. Stage IV involved distant metastasis to remote organs, which was mainly disseminated to the pleura (n = 19, 17.7%) and regional lymph nodes (n = 13, 12.1%), followed by liver, bone, kidney, brain, and adrenal gland (Table 1). Six cases showed no record of their tumor metastasis. Around 52.3% (n = 34) of the patients followed the local protocol of lung cancer treatment guidelines of surgery, radiation, and non-surgical treatments (NST). NST included different treatment modalities of single or a combination of chemotherapy (n = 43), immunotherapy (n = 4), and targeted therapy (n = 3). Out of 65 patients, 47.7% (n = 31) patients did not receive NST, however, 26 (40%) patients underwent palliative supportive treatment.

IHC for Program Death Legend-1 (*PDL-1*) expression was only tested in 10 patients. *PDL-1* was overexpressed in 4/10 (40%) patients and low expressed in 3/10 (30%) patients. Epidermal Growth Factor Receptor (*EGFR*) mutation was tested in 17 patients. *EGFR* was mutated in 5/17 (29%) patients; one patient showed exon 19 deletion and another patient had *EGFR* and Tumor Supressor-53 (*TP-53*) mutation. Other mutations detected include Anaplastic Lymphoma Kinase (*ALK*) mutation (*ALK-EML4* fusion) (n = 1), Phosphatidylinositol-4,5-Bisphosphate 3-Kinase Catalytic Subunit Alpha (*PIK3CA*) (n = 1), and two patients had isolated *TP-53* mutation (n = 3). Four patients failed to show genetic mutation due to technical lab issues. However, 3 patients showed no mutation while 49 patients refused the genetic testing. All these results are summarized in Table 1.

## Discussion

The prevalence and incidence of lung cancer in Saudi Arabia is underrated. According to SCR, two studies have estimated the incidence of lung cancer in Saudi Arabia; 452 cases diagnosed in 2014 (3.9% of all cancers per year) and 458 cases diagnosed in 2020 (3.4% of all cancers per year) [1,6]. The study conducted by Almatroudy et al., on 4530 cases in the period between 2006–2016 focused on the frequency of lung cancer by the age group [7]. The incidence of lung cancer in the Al-Madinah region of Saudi Arabia has not been accurately estimated for the past two decades. In our study, we retrospectively analyzed 65 cases of lung cancer diagnosed at the main oncology center affiliated with King Fahad Hospital in Al-Madina City in Saudi Arabia, over a period of 6 years from 2015 to 2021. We compared our findings to previous results from Surveillance, Epidemiology, and End Results (SEER) registries from 2014 to 2018 [2,3], as well as studies conducted in different racial populations, including East Asia and Europe [4]. Overall, our study found that lung cancer was more prevalent in older individuals (>50 years), Saudi males, and smokers [2]. We also observed a relatively high incidence of lung cancer in non-smokers (43%), suggesting possible exposure to secondhand smoke [2]. Additionally, 89% of the lung cancer cases were histologically diagnosed as NSCLC, which is commonly associated with smoking [2]. This finding aligned with a study by Albasri et al., who reported that lung adenocarcinoma was the most common subtype among lung cancer patients in Al-Madinah City of Saudi Arabia (47% of cases) [8].

Most studies do not show a clinically or statistically significant relationship between age, sex, and histological subtypes of lung cancers [5]. Therefore, age and sex disparities may not be determinants of the type of lung cancer [5]. The association between smoking and the development of lung cancer has been well-established, not only for smokers but also for individuals exposed to secondhand smoke [9]. However, it is important to note that not all smokers develop lung cancer, and conversely, some nonsmokers can develop lung cancer even in the absence of other environmental risk factors [9]. In a study by Che et al., it was found that smoking status alone was insufficient to explain the genomic profile differences between East Asian and European populations [10]. This suggests that there are additional factors contributing to the ancestry differences in lung cancer risk [10]. Furthermore, changes in lifestyle have led to an increase in the incidence of lung cancer among European females, which cannot be fully explained by smoking behavior alone [11]. Population-based prospective studies investigating the relationship between smoking and genetic risk in lung cancer have not been fully validated. Genome-wide association studies (GWASs) have shown that genetic factors play a lesser role in the development of lung cancer compared to environmental factors, particularly smoking [12]. Certain genes, such as CHRNA3/5, have been strongly linked to both lung cancer and smoking behaviors [13]. While previous studies have demonstrated a significant association between lung cancer and these genetic factors using case-control designs, the combined impact of these risk scores and smoking on individual subjects, as well as whether there is a synergistic effect between smoking and genetic risk, remains uncertain [14]. Zhang et al. found a strong correlation between smoking and the occurrence of lung cancer, independent of genetic risk, with the risk associated with smoking being significantly higher than that of genetic factors. This suggests that the benefits of having a low genetic risk are greatly offset by smoking, as also supported by a study conducted by Dai et al. [15,16].

There are several challenges that need to be addressed in order to achieve optimal management of lung cancer in Saudi Arabia [5]. The first challenge is the lack of a systematic approach to prevent lung cancer in primary care settings, leading to a high proportion of cases being diagnosed at late stages [5]. In our study, we found that 89% of the lung cancer cases in Al-Madinah were diagnosed at stage IV, which is consistent with the global trend of only 15% of cases being diagnosed at an early stage [2]. In Saudi Arabia, the percentage of cases diagnosed early with localized tumors is slightly lower at 14% [2]. Another challenge is the inconvenient access to cancer care facilities for patients in remote regions, as most tertiary cancer facilities are concentrated in major cities [5]. This often requires patients to travel long distances, imposing significant physical, mental, and financial burdens on both patients and their families. To address this issue, the Ministry of Health has implemented multiple projects to establish cancer facilities in smaller cities, aiming to improve accessibility to care [5]. The third challenge is related to access to medications. Many advanced chemotherapies and radiotherapies are only available for purchase at tertiary governmental hospitals, leading to prolonged hospital stays for end-of-life care [5]. Efforts are being made to overcome these challenges and improve lung cancer management in Saudi Arabia. By implementing preventive measures in primary care, expanding cancer care facilities to smaller cities, and improving access to medications, the overall management of lung cancer in the country would be enhanced [5].

Several large-scale genomic studies have extensively investigated the genomic characteristics of lung adenocarcinoma [10,17,18]. The mechanism responsible for tumor development, especially in the early stages, remains largely unknown. A study published in Nature performed by Haga et al., presented findings from whole-genome sequencing analysis of 76 lung cancers, with a focus on early-stage lung adenocarcinomas and minimally invasive adenocarcinoma [19]. The data obtained from this analysis is combined with bulk and spatial transcriptomic data as well as epigenomic data, revealing crucial events in lung carcinogenesis. In the very early stages of adenocarcinoma *in situ*, minimal somatic mutations are observed in key driver mutations and essential proliferative factors. These initial events are followed by changes in copy numbers and overall DNA hypomethylation. Significant alterations occur at later stages, specifically in Noguchi type B tumors, where cancer cells interact with the surrounding microenvironment. Key therapeutic targets, such as *EGFR* and *ALK*, have been predominantly identified. Immune checkpoint blockade (ICB) has also shown significant efficacy in patients with high expression of *PDL1* (CD274), tumor mutation burden (TMB), and gene expression profile (GEP) score [20]. It is important to note that genomic disparities exist among different racial populations. For instance, *EGFR* mutations are present in up to 60% of East Asians, whereas only 8% of Europeans exhibit these mutations [21]. In a study conducted by Chen et al., 213 lung cancer patients of Chinese descent from Singapore were sequenced, revealing that East Asians have more stable genomes characterized by fewer mutations and copy number alterations compared to individuals of European ancestry [10]. This disparity is more pronounced among smokers than nonsmokers. Additionally, nonsmoker East Asians were found to have a higher prevalence of B-Raf proto-oncogene, serine/threonine kinase (*BRAF*) mutations compared to individuals of European descent, while *EGFR* mutations were common among both smokers and nonsmokers in East Asians compared to Europeans [10].

In our study, it was found that 86% of the cases analyzed did not identify any mutations due to unsatisfactory results or patient refusal for testing [9]. However, among the cases where mutations were identified, the most common ones were *EGFR* and Tumor protein 53 (*TP53*), while *ALK* mutation was identified in a single case [9]. *PDL-1* expression was observed to be fairly expressed in seven cases [9]. These findings highlight the challenges associated with genetic testing in Saudi Arabia's healthcare system. In cases where all efforts to profile tumors, including circulating tumor DNA, have failed, the guidelines allow for the use of tyrosine kinase inhibitors (TKIs) as second- or third-line therapy [9]. Patients with driver mutations such as *EGFR*, *ALK*, and ROS Proto-Oncogene 1 (*ROS1*) are treated with targeted therapy, while patients with wild-type tumors may receive immunotherapy or systemic chemotherapy [9]. It is critical to note that all tertiary centers in Saudi Arabia have access to targeted chemotherapies such as *EGFR* TKIs (erlotinib) or *ALK* TKIs (such as crizotinib and alectinib) [9]. Early diagnosis of lung cancer has been determined as guidelines to entail detecting cancer as soon as feasible by recognizing possible early signs and symptoms and performing the necessary workup or referral to confirm or rule out malignancy. Proactive identification of cancer may be accomplished by screening asymptomatic patients for malignancy.

We acknowledge the limitations present in our study due to the small number of cases over an extended period. The number of tests conducted on the entire patient sample in our study was underestimated due to resource constraints within our institution, patient awareness limitations, and financial issues among patients.

## Conclusion

Lung cancer is not as common in Saudi Arabia as in other parts of the world. However, our study has stated that lung cancer has a significant burden on the Saudi community. If the risk factors and the challenges that we have mentioned are not addressed, the number of cases will increase drastically. Health education and awareness about the risk factors and cancer prevention methods will help in early lung cancer detection. Although significant advances in the management of this disease have been realized, there are many plans underway to enlist more resources to be better prepared to control this fatal disease through prevention and effective treatment.

## Data Availability

The datasets generated during and/or analyzed during the current study are available from the corresponding author, Yousef Katib, on reasonable request.
